# GD2-mediated impairment of macrophage phagocytosis drives pulmonary metastasis in osteosarcoma

**DOI:** 10.7150/thno.113887

**Published:** 2025-06-20

**Authors:** Yunfei He, Peng Yang, Peng Ding, Zeng Zhang, Jichuan Wu, Chunjie Wang, Guanghui Hou, Jun Ge, Quan Zhou, Zhuorun Song, Huilin Yang, Tao Liu, Shunyi Lu

**Affiliations:** 1 Department of Orthopedics, The First Affiliated Hospital of Soochow University, Suzhou 215006, China.; 2 Institutes for Translational Medicine, The First Affiliated Hospital of Soochow University, Suzhou, 215123, China.; 3 Department of Orthopedics, Shanghai Sixth People's Hospital Affiliated to Shanghai Jiao Tong University School of Medicine, Shanghai, 200233, China.; 4 Biomedical Polymers laboratory, college of chemistry chemical engineering and Materials Science and State Key laboratory of Radiation Medicine and Protection, Soochow University, Suzhou 215123, China.; 5 Institute of Functional Nano & Soft Materials (FUNSOM), Jiangsu Key Laboratory for Carbon-Based Functional Materials & Devices, Soochow University, Suzhou, 215123, China.

## Abstract

**Rationale:** The lung is the most common site of metastasis in osteosarcoma, with pulmonary dissemination accounting for most of the disease-related mortality. Despite its clinical significance, the underlying mechanisms is poorly defined.

**Methods:** To investigate the clinical relevance of GD2, we performed GD2 immunofluorescence staining on a cohort of human tumor samples. To explore the functional role of GD2 in lung metastasis, we employed an intravenous injection model and an intratibial injection model using U2OS and 143B cells respectively. To elucidate how GD2 regulates osteosarcoma lung metastasis, we carried out an* in vitro* flow-based phagocytosis assay.

**Results:** We identify the disialoganglioside GD2 as a key mediator of osteosarcoma lung metastasis through impairing macrophage phagocytic function. Mechanistically, GD2 interacts with SIGLECE in mice (or SIGLEC7 in humans) on the cell surface of macrophages, leading to the activation of SH2-containing protein tyrosine phosphatase 2 (SHP2), which in turn suppresses macrophage phagocytic function. Notably, co-treatment with an anti-GD2 antibody and the SHP2 inhibitor SHP099 resulted in a synergistic reduction of lung metastasis.

**Conclusion:** Our findings uncover a mechanism of osteosarcoma lung metastasis and highlight the GD2-SIGLEC-SHP2 axis as a promising therapeutic target.

## Introduction

Osteosarcoma is the most common primary malignant bone tumor in pediatric and adolescent populations^1^, ^2^, with a global incidence of approximately five cases per million individuals annually^3^. The current standard of care includes en-bloc surgical resection combined with neoadjuvant/adjuvant chemotherapy, with radiotherapy is typically reserved for inoperable lesions. Despite these multimodal therapeutic advances, the 5-year overall survival rate remains below 70%^4^. Alarmingly, over 15% of patients develop lung metastasis^5^, which is associated with a dramatic reduction in 5-year survival to less than 25%^6^. These sobering statistics underscore the critical unmet need for novel therapeutic strategies that target metastatic osteosarcoma.

In recent years, numerous studies have highlighted that activating osteosarcoma-infiltrating immune cells may be pivotal for effective tumor elimination^7^. Among these, macrophages, key cellular constituents of the metastatic niche, exhibit functional plasticity in tumor progression^8^-^10^. Upon stimulated by interferon-γ (IFN-γ) or microbial products, macrophages can polarize toward a classically activated M1-like phenotype^11^. These proinflammatory M1 macrophages produce high levels of cytokines such as interleukin-1 (IL-1), interleukin-12 (IL-12) as along with reactive oxygen species enabling them to directly kill tumor cells. Additionally, M1 macrophages can impede tumor progression indirectly by recruiting other immune cells including natural killer (NK) cells and T cells^12^. Conversely, M2-like macrophages are associated with tissue repair and angiogenesis and are characterized by the secretion of immunosuppressive cytokines such as interleukin-10 (IL-10) and transforming growth factor-β (TGF-β)^13^. In most malignancies, macrophages predominantly exhibit the M2-like phenotype, which can promote tumor cell growth both directly or indirectly^14^, ^15^, while also shielding tumor cells from immune attack^16^, ^17^. The role of macrophages in osteosarcoma remains controversial. Some studies have reported that tumor-associated macrophages (TAMs) promote osteosarcoma progression by suppressing T cell function^18^ and facilitating lung metastasis through the COX-2/STAT3 signaling axis^19^. Conversely, other findings suggest that macrophages may be associated with suppression of metastasis in high-grade osteosarcoma^20^, ^21^. These conflicting observations highlight the need for further investigation into the functional role of macrophages in osteosarcoma pathogenesis.

The SIGLECs, a family of sialic-acid-binding immunoglobulin-like lectins, are abundantly expressed in immune cells, especially in macrophages^22^. They are reported to modulate functions of immune cells via glycan recognition^23^. There are thirteen SIGLEC family members in humans while only nine in mice. The intracellular domains of most SIGLECs have immunoreceptor tyrosine-based inhibitory motifs (ITIMs) and signal negatively through tyrosine phosphatases such as the SH2 domain-containing protein tyrosine phosphatases SHP1 or SHP2^22^, ^23^. Recently, the neuroblastoma-expressed GD2 was identified as a ligand of SIGLEC7, which could suppress macrophage phagocytic function^24^. The disialoganglioside GD2 is a sialylated glycosphingolipid and its biosynthesis of GD2 commences with the formation of a β-linked glucose ceramide core (GlcCer) in the Golgi apparatus, followed by elongation by several glycosyltransferases, including ST3GAL5 (GM3 synthase), ST8SIA1 (GD3 synthase) and B4GALNT1 (GD2 synthase)^25^-^27^.

GD2 is aberrantly overexpressed in various malignancies, while its expression in normal tissues is minimal and largely confined to a limited number of cell types^28^, ^29^. The differential expression coupled with its potential immunomodulatory functions, makes GD2 a promising target for cancer immunotherapy. Although several GD2-targeted therapeutic strategies have been developed^30^-^33^, the precise role of GD2 in tumor progression—particularly in the context of metastasis—remains inadequately understood.

Our study elucidates a hitherto unobserved immune evasion mechanism in osteosarcoma, wherein GD2 engages SIGLECE in mice or SIGLEC7 in human on the surface of macrophage, triggering SHP2-mediated suppression of phagocytic activity. Notably, the combined inhibition of SHP2 alongside GD2 targeting synergistically enhances macrophage-mediated tumor clearance *in vitro* and significantly reduces pulmonary metastasis *in vivo*. These findings establish the GD2-SIGLEC-SHP2 axis as a promising therapeutic target for metastatic osteosarcoma.

## Results

### GD2 expression correlates with lung metastasis in osteosarcoma

**Figure 1:**
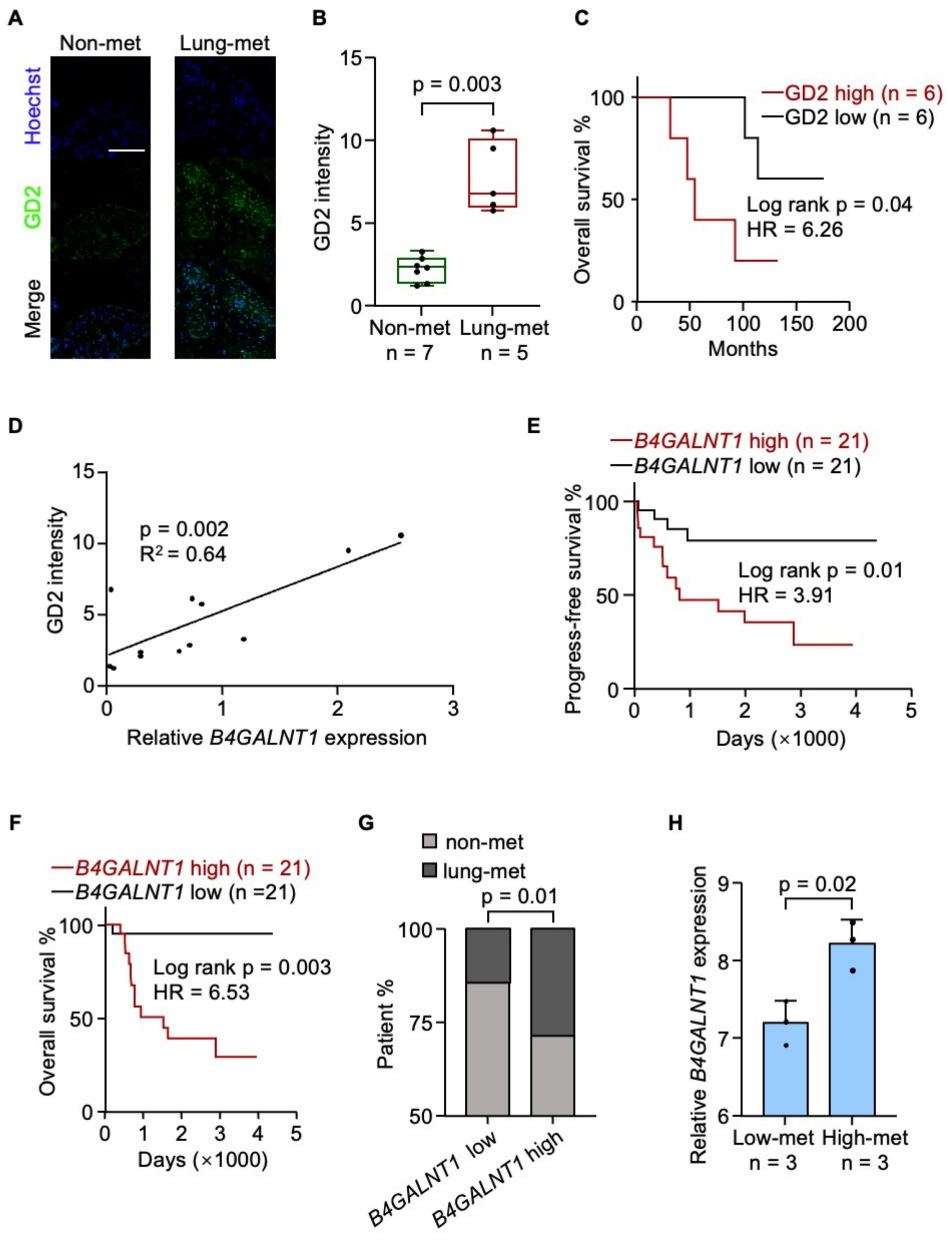
GD2 is associated with poor prognosis of osteosarcoma. (**A-C**) Analysis of GD2 expression in patient samples. Shown are presentative images (**A**), quantitation of GD2 immunostaining of osteosarcoma patient samples with or without lung metastasis (**B**) and overall survival analyses of the patients according to different GD2 expression status (**C**). (**D**) Correlation between *B4GALNT1* expression and GD2 expression in patient samples. (**E-G**) Analysis of *B4GALNT1* expression in GDC TARGET-OS dataset. Shown are progress-free survival (**E**), overall survival (**F**) analyses of the patients according to different B4GALNT1 expression status and lung metastasis rate between patients with low *B4GALNT1* expression and high *B4GALNT1* expression (**G**). (**H**) *B4GALNT1* expression in osteosarcoma cells with different metastasis potential in GSE18947 dataset. Scale bars: 100 μm. P values were obtained by log-rank test (**C**, **E** and **F**), chi-squared test (**G**), Pearson's correlation analysis (**D**) and 2-tailed unpaired t test (**B, H**). Box plots display values of minimum, first quartile, median, third quartile, and maximum. Data are represented as mean ± SD.

To investigate the clinical relevance of GD2, we performed GD2 immunofluorescence staining on a cohort of human tumor samples and observed that GD2 expression was significantly upregulated in metastatic lesions (Figure 1A and 1B). Moreover, elevated GD2 expression was associated with reduced patient survival (Figure 1C). Given that β-1,4-N-Acetyl-galactosaminyltransferase 1 (B4GALNT1) the key enzyme involved in GD2 iosynthesis^34^ and showed a strong positive correlation with GD2 expression (Figure 1D), we further examined its clinical relevance using the TARGET-OS dataset. Elevated *B4GALNT1* expression was significantly correlated with poorer overall survival (Figure 1E and 1F) and an increased risk of lung recurrence (Figure 1G). In line with this findings, high-metastatic osteosarcoma cell lines also exhibited upregulated *B4GALNT1* expression (GSE18947; Figure 1H). These findings support a strong association between GD2 and lung metastasis in osteosarcoma.

### GD2 promotes lung metastasis of osteosarcoma

**Figure 2:**
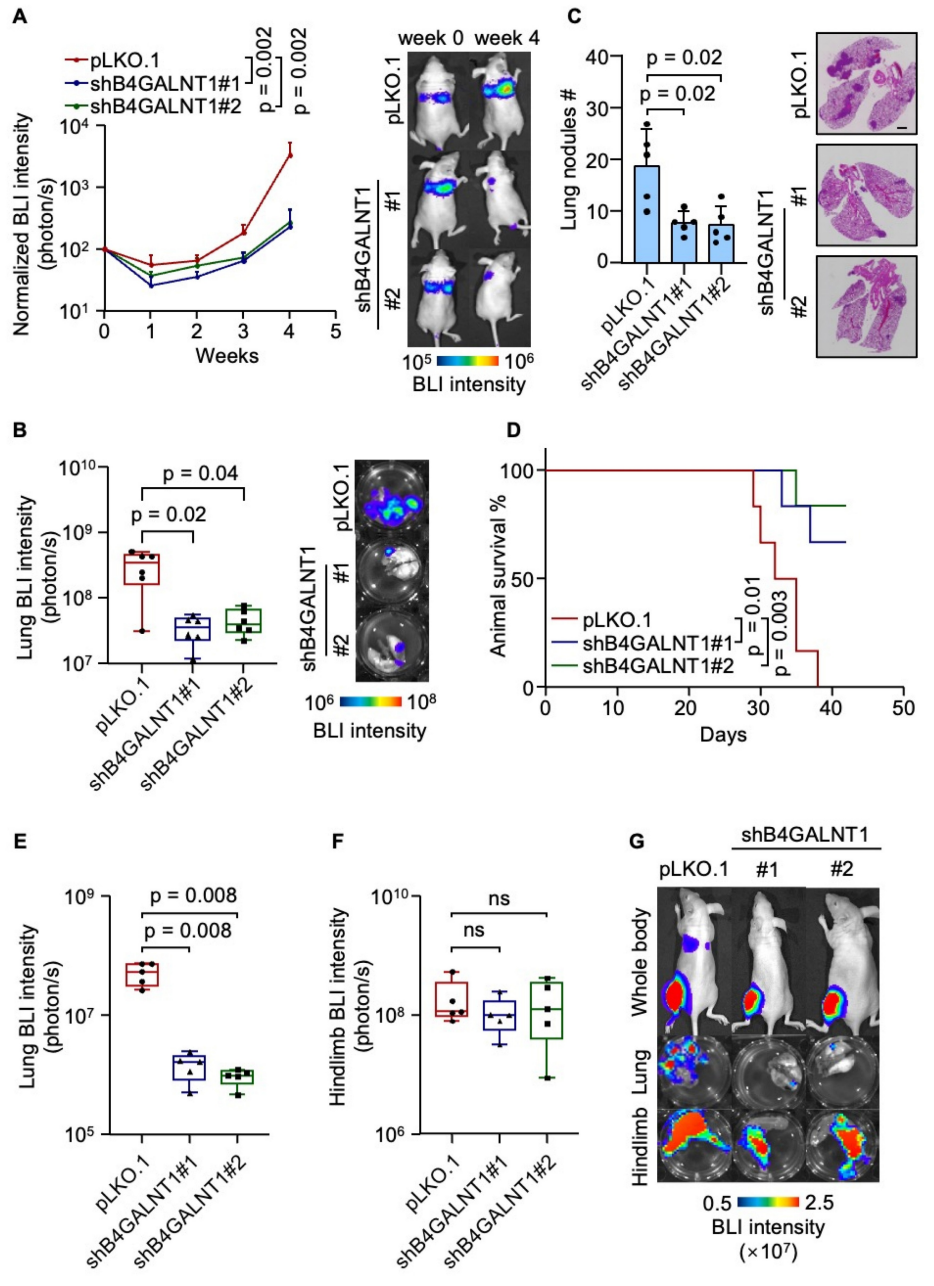
GD2 promotes lung metastasis of osteosarcoma. (**A-D**) Intravenous injection of U2OS with or without *B4GALNT1* knockdown into nude mice for lung metastasis analysis. Weekly BLI quantitation of tumor burden of the mice and representative images (**A**, n = 6 mice per group), *ex vivo* BLI analysis of lungs and representative images (**B**), number of lung nudes and representative images (**C**) and animal survival (**D**, n = 6 mice per group). (**E-G**) Intratibial injection of 143B with or without *B4GALNT1* knockdown into nude mice for lung metastasis analysis.* Ex vivo* BLI analysis of lungs (**E**) and hindlimbs (**F**) at week 6 and representative images (**G**). Scale bars: 3 mm. P values were obtained by Mann-Whitney U test (**A**,** B**,** E** and **F**), 2-tailed unpaired t test (**C**) and log-rank test (**D**). Box plots display values of minimum, first quartile, median, third quartile, and maximum. Data are represented as mean ± SD.

To explore the functional role of GD2 in lung metastasis, we established GD2 depleted U2OS cell lines by knocking down *B4GALNT1* (Figure S1A-S1C). Then, we intravenously inoculated the U2OS cells with or without GD2 depletion, respectively, into immunodeficient mice and metastatic burden was assessed by bioluminescent imaging (BLI). Weekly BLI monitoring showed that GD2 depletion markedly reduced metastatic burden (Figure 2A). *Ex vivo* analyses lung tissues revealed an approximate 10-fold reduction of metastatic burden by week four in the GD2-depleted groups (Figure 2B). This was further supported by a marked decrease in the number of lung metastatic nodules (Figure 2C) and corresponding improvement in overall survival time (Figure 2D). To validate these findings in a clinically relevant model. we employed an orthotopic osteosarcoma metastasis model^35^ using 143B cells with or without GD2 depletion (Figure S1D-S1F), injected into the tibia of mice. GD2 depletion significantly suppressed spontaneous lung metastasis of 143B tumors to lungs (Figure 2E and 2G) while the primary tumor growth remained unaffected (Figure 2F and 2G), suggesting that the effect of GD2 on metastasis may be context-depended and influenced by the tumor microenvironments. Collectively, these results demonstrate a pro-metastatic role for GD2 in osteosarcoma within immunodeficient mouse model.

### GD2 impairs macrophage phagocytic function

**Figure 3:**
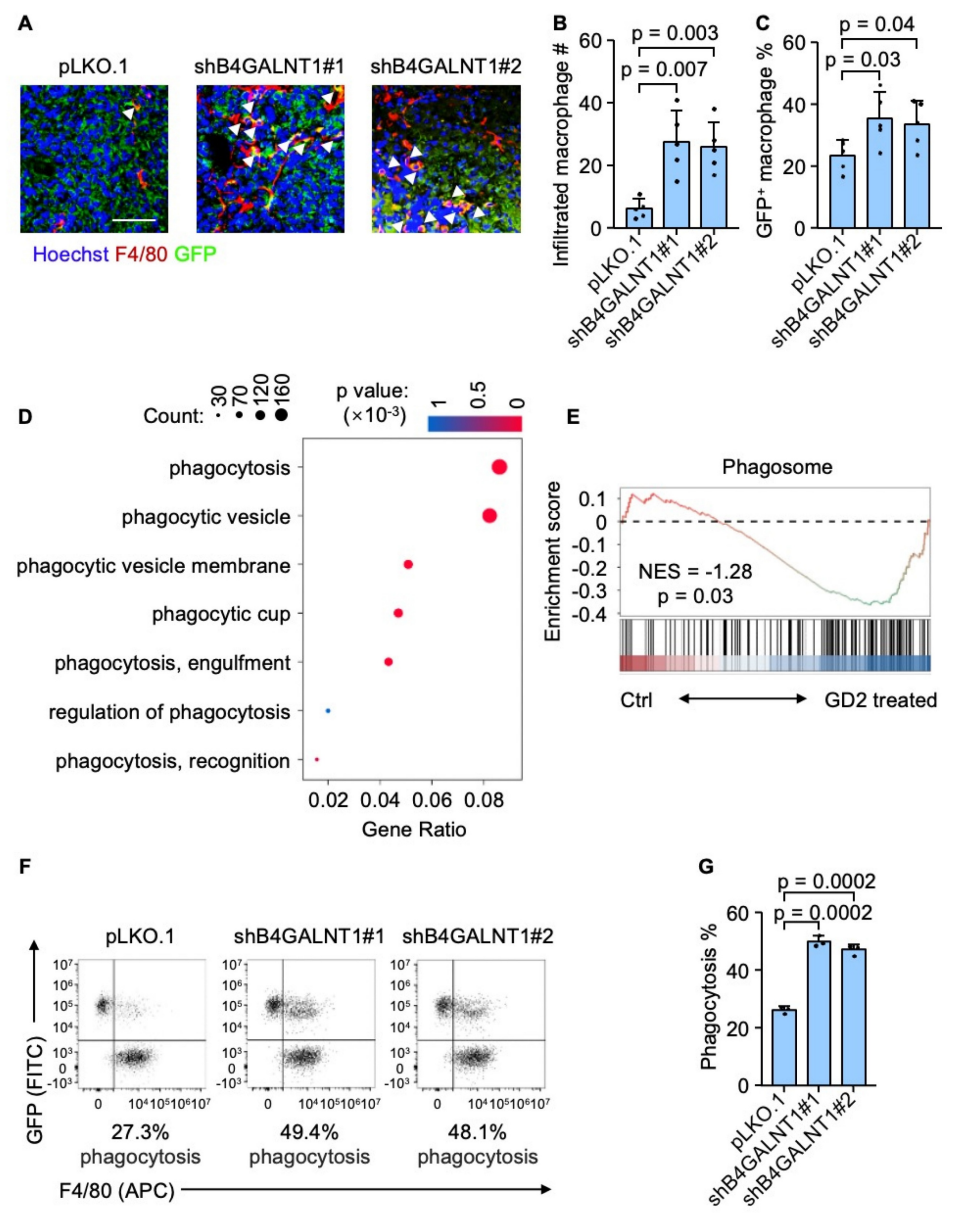
GD2 suppresses phagocytosis of osteosarcoma cells. (**A**-**C**) Immunofluorescence analyses of lung metastasis niches at day 28 after intravenous injection of U2OS cells (with or without *B4GALNT1* knockdown). Shown are representative images (**A**), quantitation of tumor infiltrated macrophages (**B**) and percentage of GFP^+^ macrophages (**C**). (**D**, **E**) GO analyses of enriched genes (**D**) in the RNA-seq profiles of GD2-treated mouse bone marrow derived macrophages vs control mouse bone marrow derived macrophages and GSEA analyses of Phagosome gene sets (**E**). (**F**, **G**) Representative flow cytometry plots depicting the phagocytosis of GFP-labeled U2OS cells (with or without *B4GALNT1* knockdown) co-cultured with mouse bone marrow derived macrophages (**F**) and flow-cytometry-based quantification of phagocytosis of U2OS in the presence of mouse bone marrow derived macrophages (**G**). Scale bars: 50 μm. P values were obtained by 2-tailed unpaired t test (**B**, **C** and **G**). Data are represented as mean ± SD.

To further elucidate how GD2 regulates osteosarcoma lung metastasis, we focused on the role of tumor-infiltrating macrophages, which are key components of the metastatic niche in the lung. We hypothesized that GD2 might promote lung metastasis by regulating macrophage function within the tumor microenvironment. Then we carried out an immunostaining analysis of murine lung metastatic foci revealed a significant increase in tumor-infiltrating macrophage following GD2 depletion (Figure 3A and 3B). Moreover, there was a notable rise in GFP-positive macrophages indicating enhanced phagocytosis of GFP-labeled tumor cells by macrophages (Figure 3A and 3C). To further verify whether GD2 directly suppress macrophage-mediated phagocytosis, we performed a bulk RNA sequencing of murine bone marrow-derived macrophages (BMDMs). Consistent with the above findings, gene ontology (GO) analysis and gene set enrichment analysis (GSEA) revealed a downregulation of phagocytosis-related pathways in GD2-treated macrophages (Figure 3D and 3E). In addition, an *in vitro* phagocytosis assay also demonstrated that GD2 depletion promoted the phagocytosis of osteosarcoma cells by murine BMDMs (Figure 3F and 3G) and THP-1 derived macrophages (Figure S2). These findings indicate that GD2 facilitates osteosarcoma lung metastasis by suppressing macrophage-mediated phagocytosis within the TME.

### SIGLECE/7 mediates GD2's anti-phagocytic signaling

**Figure 4:**
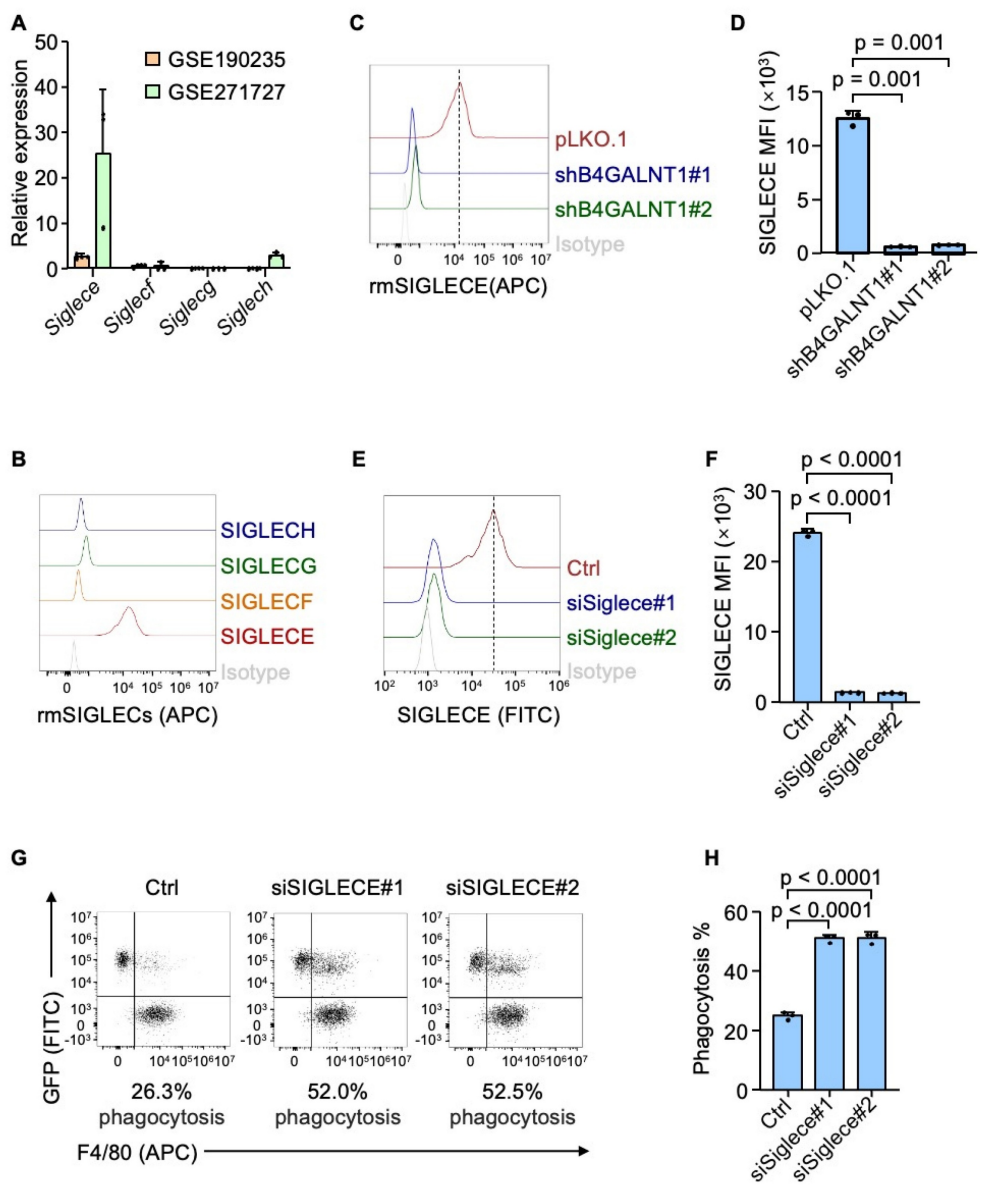
GD2 functions by interacting with SIGLECs. (**A**) *Siglece, Siglecf, Siglecg and Siglech* expression in mouse bone marrow derived macrophages in GSE190235 and GSE271727 datasets. (**B**) Flow cytometric histograms of U2OS stained with recombinant mouse SIGLECs. (**C**) Flow cytometric histograms of U2OS (with or without *B4GALNT1* knockdown) stained with recombinant mouse SIGLECE. (**D**) Quantitation of SIGLECE mean fluorescence intensity (MFI) in panel (**C**). (**E**) Flow cytometric analysis of SIGLECE expression in mouse bone marrow derived macrophages (with or without *Siglece* knockdown). (**F**) Quantitation of SIGLECE mean fluorescence intensity (MFI) in panel (**E**). (**G**, **H**) Representative flow cytometry plots depicting the phagocytosis of GFP-labeled U2OS cells co-cultured with mouse bone marrow derived macrophages (with or without *Siglece* knockdown) (**G**) and flow-cytometry-based quantification of phagocytosis of U2OS in the presence of mouse bone marrow derived macrophages (**H**). P values were obtained by 2-tailed unpaired t test (**C**, **E** and **G**). Data are represented as mean ± SD.

GD2 is a sialylated glycolipid that belongs to sialoglycan family, which are known to interact with SIGLECs (sialic acid-binding immunoglobulin-like lectins) - a group of immunoregulatory receptors predominantly expressed immune cells, including macrophages^36^. Particularly, GD2 could interact with SIGLEC7 in human neuroblastoma^24^. Based on this, we hypothesized that GD2 may exert its immunosuppressive function through binding to SIGLEC7. We confirmed the interaction between GD2 and SIGLEC7 in osteosarcoma by flow cytometry (Figure S3A and S3B). Furthermore, the knocking down *SIGLEC7* in THP-1 derived macrophages dramatically enhanced their phagocytosis of osteosarcoma cells (Figure S3C-S3F). With promising in vitro results, we continue to analyze the interaction between GD2 and SIGLEC7 in patient samples using immunostaining assay. Immunofluorescence staining revealed clear co-localization of GD2 and SIGLEC7 (Figure S3G), validating their association in the clinical setting. However, SIGLEC7 is exclusively expressed in human cells and lacks a direct homolog in mice. To identify the relevant murine receptor for GD2, we analyzed public datasets (GSE190235 and GSE271727) and identified that among the four mouse-specific Siglecs (*Siglece*, *Siglecf*, *Siglecg* and *Siglech*), *Siglece* was most highly expressed murine BMDMs murine BMDMs (Figure 4A). Flow cytometry analysis using recombinant proteins confirmed that SIGLECE exhibited the strongest binding to U2OS osteosarcoma cells (Figure 4B). Importantly, genetic ablation of GD2 markedly reduced the binding of SIGLECE to U2OS cells (Figure 4C and 4D). Moreover, knockdown of *Siglece* in murine BMDMs significantly enhanced phagocytosis of osteosarcoma cells (Figure 4E-4H). These findings suggest that GD2 interacts with SIGLEC7 in humans and SIGLECE in mice to transmit an inhibitory "don't eat me" signal from osteosarcoma cells to macrophages, thereby promoting immune evasion.

### GD2 activates SH2-containing protein tyrosine phosphatase 2 (SHP2) through SIGLECE

**Figure 5:**
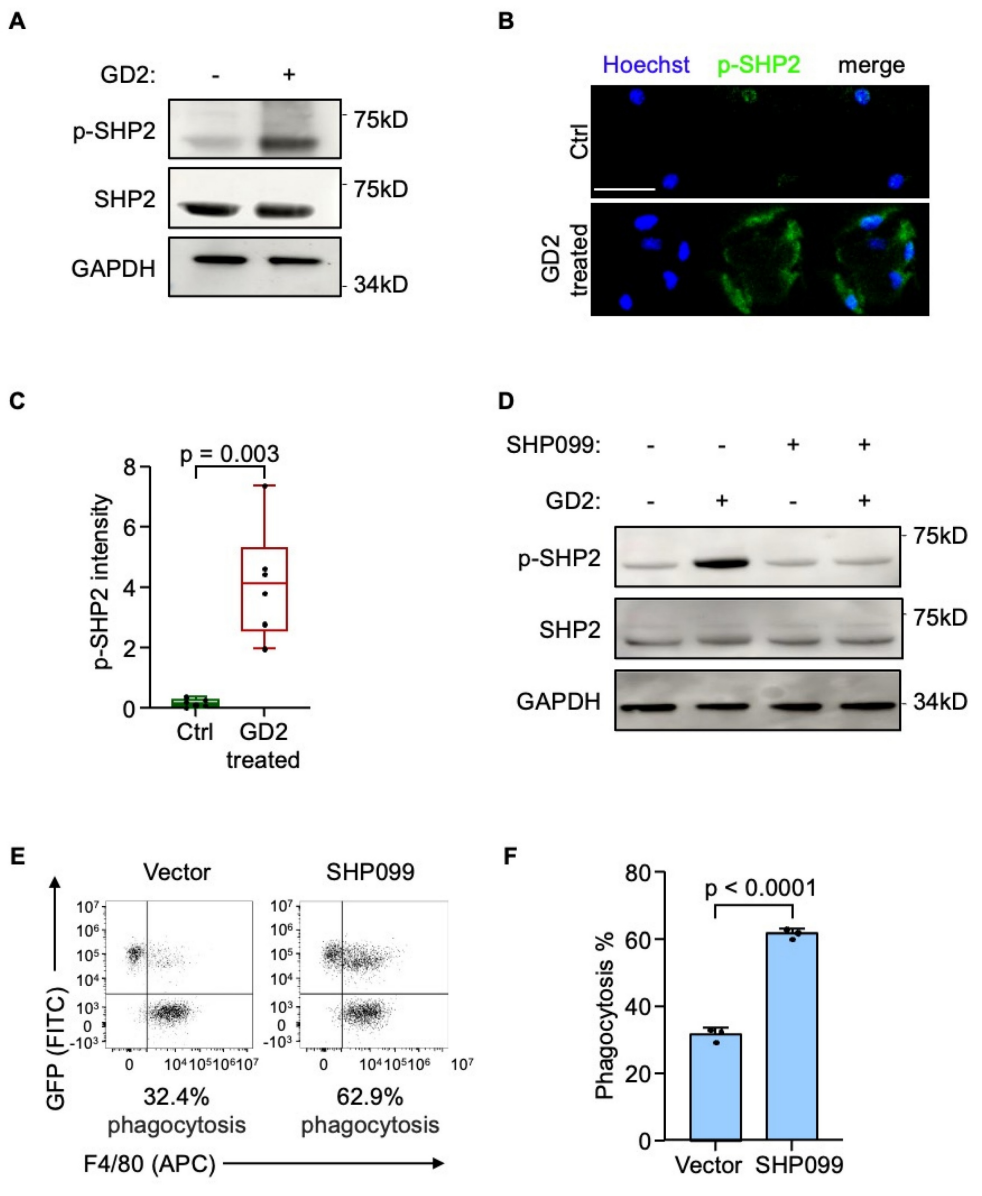
GD2 activates SH2-containing protein tyrosine phosphatase 2. (**A**) Western blot analysis of phosphorylated SHP2 protein level in mouse bone marrow derived macrophages after treated with GD2. (**B**,** C**) Representative images (**B**) and quantitation (**C**) of phosphorylated SHP2 immunostaining of mouse bone marrow derived macrophages after treated with GD2. (**D**) Western blot analysis of phosphorylated SHP2 protein level in mouse bone marrow derived macrophages after treated with GD2 and/or SHP099. (**E**, **F**) Representative flow cytometry plots depicting the phagocytosis of GFP-labeled U2OS cells co-cultured with mouse bone marrow derived macrophages (with or without SHP099 treatment) (**E**) and flow-cytometry-based quantification of phagocytosis of U2OS in the presence of mouse bone marrow derived macrophages (**F**). Scale bars: 100 μm. P values were obtained by 2-tailed unpaired t test (**C** and **F**). Box plots display values of minimum, first quartile, median, third quartile, and maximum. Data are represented as mean ± SD.

Next, we sought to delineate the downstream mechanism of SIGLECE/7. The SH2-containing protein tyrosine phosphatase 2 (SHP2) was the downstream signal-transduction molecule of SIGLECE/7^23^, ^37^. Importantly, inhibition of SHP2 has been reported to enhance macrophage endocytosis^38^. Hence, we analyzed whether GD2 regulates SHP2 activation via SIGLECE/7. Treatment of murine BMDMs with GD2 led to increased phosphorylation of SHP2 (Figure 5A-5C) while knockdown of *Siglece* in murine BMDMs significantly reduced SHP2 phosphorylation (Figure S4A) supporting SIGLECE as a mediator of this effect. Furthermore, treatment with SHP099, a selective SHP2 inhibitor, effectively blocked GD2-induced SHP2 activation (Figure 5D and S4B). Importantly, pharmacologic inhibition of SHP2 markedly enhanced the phagocytic activity of macrophages toward osteosarcoma cells (Figure 5E and 5F, S4C and S4D). These results demonstrate that GD2 engages SIGLECE/SIGLEC7 on macrophages to activate downstream SHP2 signaling, thereby suppressing phagocytosis and promoting immune evasion.

### Combined SHP099 and anti-GD2 therapy suppresses osteosarcoma lung metastasis

**Figure 6:**
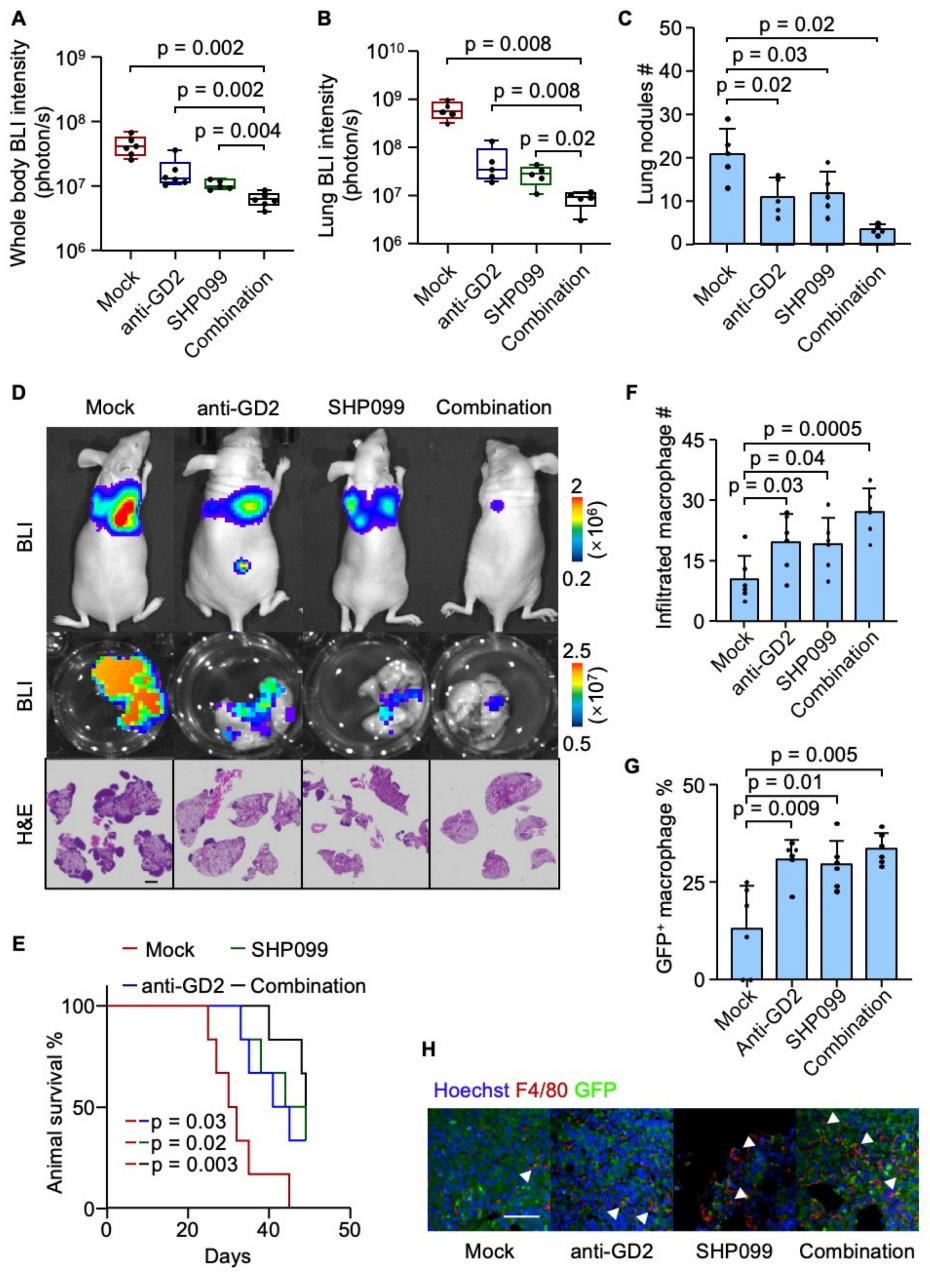
Combinatory SHP099 and Dinutuximab treatment effectively suppress lung metastasis. (**A-E**) Intravenous injection of U2OS into nude mice with different treatments for lung metastasis analysis. Whole body BLI quantitation of tumor burden of the mice at week 4 (**A**, n = 6, Mock; 6, 14G2a; 5, SHP099; 6, combination.) and representative images (**D**), *ex vivo* BLI analysis of lungs (**B**) and representative images (**D**), number of lung nudes (**C**) and representative images (**D**) and animal survival (**E**, n = 6 mice per group). (**F-H**) Immunofluorescence analyses of lung metastasis niches at day 28 after intravenous injection of U2OS cells with different treatments. Shown are quantitation of tumor infiltrated macrophages (**F**), percentage of GFP^+^ macrophages (**G**) and representative images (**H**). Scale bars: 3 mm. P values were obtained by Mann-Whitney U test (**A** and **B**), 2-tailed unpaired t test (**C, F** and** G**) and log-rank test (**E**). Box plots display values of minimum, first quartile, median, third quartile, and maximum. Data are represented as mean ± SD.

Having established the role of the GD2-SIGLECE/7-SHP2 signaling axis in suppressing macrophage-mediated phagocytosis and promoting lung metastasis in osteosarcoma, we next investigated therapeutic potential of targeting this pathway. The U2OS cells were inoculated intravenously into mice to establish the osteosarcoma lung metastasis model. One-week post-inoculation, the mice were randomized into four groups. In the anti-GD2 group, mice received intraperitoneal injections of 300 μg anti-GD2 antibody three times per week. The SHP099 group was treated with daily oral doses of 0.75 mg SHP099 per mouse, dissolved in a vehicle of 10% DMSO and 90% 20% SBE-β-CD in saline. The combination therapy group received both anti-GD2 antibody (300 μg, three times weekly) and SHP099 (0.75 mg daily). The control group (mock) was administered with 300 μg of isotype control IgG along with the SHP099 solvent. While monotherapy with either SHP099 or anti-GD2 antibody moderately suppressed metastatic burden, the combination therapy resulted in a synergistic suppression of lung metastasis and prolonged mouse survival (Figure 6A-6E). Consistent with prior findings, both treatments—individually and in combination—significantly enhanced macrophage infiltration into the tumor microenvironment and augmented the phagocytosis of tumor cell (Figure 6F-6H). These results highlight the therapeutic efficacy of targeting the GD2-SIGLECE/7-SHP2 axis to inhibit osteosarcoma lung metastasis and enhance anti-tumor immunity.

## Discussion

Osteosarcoma is the most common primary malignant bone tumor and is frequently associated with a high risk of metastasis, particularly to the lungs. Pulmonary metastasis remains the principal challenge in osteosarcoma treatment, with the five-year survival rate for affected patients remaining below 25% despite systemic chemotherapy^6^. Advances in cancer immunology have introduced immunotherapy as a promising therapeutic strategy for osteosarcoma. GD2, which is aberrantly expressed in osteosarcoma compared to normal tissues, has emerged as a potential immunotherapeutic target^28^. However, clinical trials evaluating GD2-targeted therapies in recurrent osteosarcoma have not yielded successful outcomes^39^. Consequently, it is critical to elucidate the role and underlying mechanisms of GD2 in osteosarcoma lung metastasis to facilitate the development of more effective treatment strategies. In this study, we employed two distinct immunodeficient mouse models to investigate the pro-metastatic role of GD2 in osteosarcoma. Mechanistically, GD2 was found to interact with SIGLEC-E/7 on macrophages, leading to the phosphorylation of SHP2 and subsequent suppression of macrophage-mediated phagocytosis. Moreover, both GD2 depletion and SHP2 inhibition significantly reduced the incidence of lung metastasis. Notably, GD2 depletion did not influence primary tumor growth, which may be attributed to differences in the tumor microenvironment between primary and metastatic sites. Unlike the lung metastatic niche, the primary tumor microenvironment uniquely contains osteoclasts. Osteoclasts have been implicated as pro-tumorigenic components not only in primary osteosarcoma^40^ but also in other bone tumors^41^ and bone-metastatic cancers^42^.

In addition, we identified SIGLECE as a GD2 receptor in mice. While SIGLEC7 has been recognized as the primary GD2 receptor in humans, the lack of a murine homolog introduces uncertainty regarding the extent to which osteosarcoma xenograft models accurately reflect human disease pathology. To address this, we conducted a flow cytometry-based screening using recombinant proteins corresponding to four mouse-specific SIGLECs and confirmed SIGLECE as a GD2-binding receptor in mice. Furthermore, both SIGLECE and SIGLEC7 were capable of inducing SHP2 phosphorylation and suppressing macrophage phagocytic activity. Collectively, these findings indicate that SIGLECE serves as a functional equivalent of human SIGLEC7 in the murine system.

Our findings also support SHP2-targeted therapy as a potential strategy to treat osteosarcoma lung metastasis. A previous clinical trial (NCT02484443) evaluating the combination of dinutuximab, a GD2-specific antibody, with GM-CSF did not demonstrate a significant improvement in disease control rates in patients with recurrent osteosarcoma^39^. Based on the trial outcomes and the investigators' conclusions, we hypothesize that the lack of GD2 expression screening among enrolled patients may have contributed to the limited efficacy observed. This likely resulted in the inclusion of patients with low or absent GD2 expression, a subgroup inherently less responsive to dinutuximab-mediated therapy. Furthermore, dinutuximab primarily exerts its effects through antibody-dependent cellular cytotoxicity (ADCC), which may be insufficient in the context of an immunosuppressive tumor microenvironment. In our preclinical studies, SHP2 inhibition using SHP099 was shown to alleviate this immunosuppressive milieu by promoting macrophage-mediated phagocytosis, thereby enhancing antitumor responses. Additionally, SHP2 deletion has been reported to skew myeloid cell differentiation toward proinflammatory phenotypes and enhance their antigen-presenting capacity^43^. Collectively, these findings suggest that SHP2 inhibition may serve as a complementary therapeutic approach, particularly for patients who do not benefit from GD2-targeted therapies.

Several limitations should be considered when interpreting our findings. First, our conclusions are based on studies conducted in immunodeficient Balb/c nude mice, which possess a congenital thymic defect resulting in impaired T cell development, while other immune cell populations, including macrophages, are largely preserved^44^. Although macrophages are theoretically functional in these models, T cell-macrophage crosstalk may be essential for fully recapitulating macrophage activity^45^, ^46^. Therefore, the absence of T cells could indirectly affect macrophage function, underscoring the need for further validation in immunocompetent mouse models to ensure translational relevance. However, current commercial GD2 antibodies do not cross-react with murine GD2, making it difficult to detect GD2 expression in mouse osteosarcoma cells and limiting the feasibility of such studies using syngeneic models. This limitation highlights the need to develop appropriate humanized mouse models. Also, in the *in vivo* experiments, although the animal survival was prolonged by GD2 or SHP2 blockade, the mice became debilitated at the study endpoint. This outcome is most likely attributable to the fact that mice across all groups developed lung metastases to varying extents. The control group mice, suffering from more severe lung metastases, perished first. Though the knockdown group and drug-treated group mice demonstrated a certain level of survival, their lung metastases progressively deteriorated, leaving them in an extremely weak state. Additionally, our data indicates that GD2 primarily suppresses macrophage-mediated phagocytosis, suggesting that the degree of macrophage infiltration within the tumor microenvironment may influence patient responsiveness to GD2-targeted therapies. Thus, the density of tumor-infiltrating macrophages could serve as a potential biomarker for predicting therapeutic outcomes. Nevertheless, further investigation and clinical validation are required to confirm this hypothesis.

## Conclusion

Overall, the present study significantly advances our understanding of the functional role of GD2 in promoting lung metastasis in osteosarcoma. By elucidating the mechanistic interaction between GD2 and the SIGLEC-E/SHP2 axis in macrophages, our findings provide important insights into the immunosuppressive pathways that facilitate metastatic progression. Furthermore, the study highlights the therapeutic potential of targeting the GD2 signaling axis, not only as a direct antitumor strategy but also as a means to modulate the tumor immune microenvironment. These findings lay the groundwork for the development of more effective, immune-based interventions for metastatic osteosarcoma and underscore the importance of further translational research to optimize and validate GD2-targeted therapies in clinical settings.

## Materials and Methods

### Cell culture

U2OS, 143B and THP-1 cell lines were purchased from the Cell Bank of Type Culture Collection of the Chinese Academy of Sciences. Both U2OS and THP-1 were cultured in RPMI-1640 supplemented with 10% fetal bovine serum (JYK-FBS-301, Jin Yuan Kang Biotechnology), penicillin (100 units/mL) and streptomycin (100 μg/mL). 143B was cultured in MEM-EBSS supplemented with 10% fetal bovine serum (JYK-FBS-301, Jin Yuan Kang Biotechnology), penicillin (100 units/mL) and streptomycin (100 μg/mL).

### Constructs and reagents

The annealed sense and antisense shRNA oligonucleotides were cloned into the pLKO.1-puro vector (Addgene) for knockdown of human *B4GALNT1* with the following target sequences: GCTGCTTTCACTATCCGCATA (sh*B4GALNT1*#1), CAACTACAACTGGTCACTTAC (sh*B4GALNT1*#2). The siRNAs for knockdown of *SIGLEC7* are: GAGTCCTGGGCAGATGATAAC (si*SIGLEC7*#1), CATAGGCATGAAGGATGCAAA (si*SIGLEC7*#2). The siRNAs for knockdown of *Siglece* are: CCCAATTCGTAAAGCAGTGAA (si*Siglece*#1), GCCACAAATAACCCAATTCGT (si*Siglece*#2). The antibodies used for Western blotting, flow cytometry and immunofluorescence were as follows: GD2-APC (357305, Biolegend for flow cytometry), GD2 (sc-101351, Santa Cruz for immunofluorescence; BE0318, BioXCell for *in vivo* treatment), SHP2 (ab300579, Abcam), phosphor-SHP2 (ab62322, Abcam), GAPDH (ZB15004-HRP-100, Servicebio), GFP (ab13970, abcam), F4/80 (ab111101, Abcam for immunofluorescence), F4/80-APC (123115, Biolegend for flow cytometry), CD68-APC (333809, Biolegend), SIGLECE-FITC (677111, Biolegend), SIGLEC7-FITC (MA5-28199, Invitrogen for flow cytometry), SIGLEC7 (abs102726, absin). The SHP2 inhibitor SHP099 for was obtained from MedChemExpress (HY-100388).

### Siglec ligand detection

Siglec ligands were quantified via flow cytometry. First, 4 μg/mL of each SIGLEC-Fc (obtained from R&D Systems: SIGLECE-Fc, 5806-SL; SIGLECF-Fc, 1706-SF; SIGLECG-Fc, 10103-SL; SIGLECH-Fc, 10264-SH; SIGLEC7-Fc, 1138-SL) was pre-complexed with 8 μg/mL of AffiniPure goat anti-human IgG Alexa Fluor 647 (109-605-008, JacksonImmunoResearch), or 8 μg/mL of AffiniPure™ goat anti-mouse IgG Alexa Fluor 647 (115-605-008, JacksonImmunoResearch) in FACS buffer (3% fetal bovine serum in PBS) for 30min on ice. Cells were washed twice with FACS buffer and then resuspended at 4 × 10^6^ cells per milliliter.

### Macrophage differentiation and transfection

For THP-1 cells, they were harvested and the density was adjusted to 5×10^5^ cells/mL. Then the cells were stimulated with 100 ng/mL PMA in 6-well plates (801004, NEST) for 48 h. Next, the cells were further treated with 20 ng/mL IFN-γ (CM40, novoprotein) and 240 ng/mL LPS for 48 hours. For murine bone marrow cells, they were seeded as 5×10^5^ cells/mL and were stimulated with 50 ng/mL M-CSF (CR97, novoprotein) in 10-cm dishes (704202, NEST) for 7 days. Next, the cells were further treated with 200 ng/mL IFN-γ (CI57, novoprotein) and 1 μg/mL LPS for 48 hours. Lipofectamine 2000 (11668019, Thermo Fisher Scientific) was used to transfect siRNAs when the differentiation was completed. The follow-up experiments were carried out 36 hours after transfection. All cells were cultured in ultra-low-attachment 60-mm-disher (FULA060, Beyotime).

### *In vitro* flow-based phagocytosis assay

GFP-labeled tumor cells and macrophages were co-cultured at a ratio of 2:1 in ultra-low-attachment 96-well U-bottom plates (FULA962, Beyotime) in serum-free RPMI-1640 for 4hours at 37 °C. Next, the cells were stained with anti-F4/80 (for BMDM) or anti-CD68 (for THP-1 derived macrophage). Assays were analyzed by flow cytometry, and phagocytosis was measured as the ratio of F4/80^+^/ CD68^+^ and GFP^+^ macrophages to total F4/80^+^/ CD68^+^ macrophages.

### Transcriptomic sequencing and data analysis

The RNA of BMDMs treated with 200 μM GD2 (HY-165740, MedChemExpress) or PBS (as control) were used for transcriptome sequencing. The sequencing data was analyzed by the following R packages: DESeq2, clusterProfiler and fgsea. The sequencing data was available in NODE (https://www.biosino.org/node) with the accession number OEP00006179.

### Mouse experiments

All animal studies were conducted according to the guidelines for the care and use of laboratory animals and were approved by Soochow University. Mice were randomly grouped with approximately equal body weight between groups. No mice were excluded from analyses except those with unexpected death from non-tumor reason. Investigators were not blinded to allocation during the experiments and outcome assessment. For lung metastasis analysis, 1×10^6^ tumor cells were intravenously injected into nude mice. BLI data were acquired with an IVIS Spectrum CT system (PerkinElmer). For orthotopic osteosarcoma metastasis analysis, exponentially growing osteosarcoma cells were harvested, counted, and resuspended in PBS to a final concentration of 10^7^ cells/mL. About 1×10^6^ cells in 100μL of PBS were injected into the left tibia. After six weeks, lung metastases were assessed using bioluminescence imaging.

### Clinical analysis

The osteosarcoma cohort, consisting of 12 human primary osteosarcoma tissues and 2 lung metastasis tissues were obtained from the First Affiliated Hospital of Soochow University. Informed consent from all participants and approval from the Hospital's Research Ethics Committee were obtained for tissue retrieval. GD2 was immune-stained and the staining intensities were quantified using Image J (NIH).

### Statistical analyses

Data analyses were performed using GraphPad Prism 8.0 (GraphPad Software, La Jolla, USA). The data presentation and statistical analyses are described in the figure legends. P values < 0.05 were considered as statistically significant. The experiments *in vitro* were repeated independently multiple times with similar results, as indicated in the figure legends.

## Supplementary Material

Supplementary figures.

## References

[1] Mirabello L, Troisi RJ, Savage SA (2009). Osteosarcoma incidence and survival rates from 1973 to 2004: data from the Surveillance, Epidemiology, and End Results Program. Cancer.

[2] Pingping B, Yuhong Z, Weiqi L, Chunxiao W, Chunfang W, Yuanjue S (2019). Incidence and Mortality of Sarcomas in Shanghai, China, During 2002-2014. Front Oncol.

[3] Mirabello L, Troisi RJ, Savage SA (2009). International osteosarcoma incidence patterns in children and adolescents, middle ages and elderly persons. Int J Cancer.

[4] Smeland S, Bielack SS, Whelan J, Bernstein M, Hogendoorn P, Krailo MD (2019). Survival and prognosis with osteosarcoma: outcomes in more than 2000 patients in the EURAMOS-1 (European and American Osteosarcoma Study) cohort. Eur J Cancer.

[5] Kager L, Zoubek A, Pötschger U, Kastner U, Flege S, Kempf-Bielack B (2003). Primary metastatic osteosarcoma: presentation and outcome of patients treated on neoadjuvant Cooperative Osteosarcoma Study Group protocols. J Clin Oncol.

[6] Gill J, Gorlick R (2021). Advancing therapy for osteosarcoma. Nat Rev Clin Oncol.

[7] Lu S, Yang Y, Song Z, Cao J, Han Z, Chen L (2025). Dual functional nanoplatforms potentiate osteosarcoma immunotherapy via microenvironment modulation. National Science Review.

[8] Qian BZ, Pollard JW (2010). Macrophage diversity enhances tumor progression and metastasis. Cell.

[9] Quail DF, Joyce JA (2013). Microenvironmental regulation of tumor progression and metastasis. Nat Med.

[10] Zhuang X, Zhang H, Hu G (2019). Cancer and Microenvironment Plasticity: Double-Edged Swords in Metastasis. Trends Pharmacol Sci.

[11] Mosser DM, Edwards JP (2008). Exploring the full spectrum of macrophage activation. Nat Rev Immunol.

[12] Shapouri-Moghaddam A, Mohammadian S, Vazini H, Taghadosi M, Esmaeili SA, Mardani F (2018). Macrophage plasticity, polarization, and function in health and disease. J Cell Physiol.

[13] Yunna C, Mengru H, Lei W, Weidong C (2020). Macrophage M1/M2 polarization. Eur J Pharmacol.

[14] Zhu R, Huang J, Qian F (2025). The role of tumor-associated macrophages in lung cancer. Front Immunol.

[15] Munir MT, Kay MK, Kang MH, Rahman MM, Al-Harrasi A, Choudhury M (2021). Tumor-Associated Macrophages as Multifaceted Regulators of Breast Tumor Growth. Int J Mol Sci.

[16] Tharp KM, Kersten K, Maller O, Timblin GA, Stashko C, Canale FP (2024). Tumor-associated macrophages restrict CD8(+) T cell function through collagen deposition and metabolic reprogramming of the breast cancer microenvironment. Nat Cancer.

[17] Nixon BG, Kuo F, Ji L, Liu M, Capistrano K, Do M (2022). Tumor-associated macrophages expressing the transcription factor IRF8 promote T cell exhaustion in cancer. Immunity.

[18] Han Q, Shi H, Liu F (2016). CD163+ M2-type tumor-associated macrophage support the suppression of tumor-infiltrating T cells in osteosarcoma. International Immunopharmacology.

[19] Han Y, Guo W, Ren T, Huang Y, Wang S, Liu K (2019). Tumor-associated macrophages promote lung metastasis and induce epithelial-mesenchymal transition in osteosarcoma by activating the COX-2/STAT3 axis. Cancer Letters.

[20] Buddingh EP, Kuijjer ML, Duim RA, Bürger H, Agelopoulos K, Myklebost O (2011). Tumor-infiltrating macrophages are associated with metastasis suppression in high-grade osteosarcoma: a rationale for treatment with macrophage activating agents. Clin Cancer Res.

[21] Zhao Y, Zhang B, Zhang Q, Ma X, Feng H (2021). Tumor-associated macrophages in osteosarcoma. J Zhejiang Univ Sci B.

[22] Crocker PR, Paulson JC, Varki A (2007). Siglecs and their roles in the immune system. Nature Reviews Immunology.

[23] Macauley MS, Crocker PR, Paulson JC (2014). Siglec-mediated regulation of immune cell function in disease. Nat Rev Immunol.

[24] Theruvath J, Menard M, Smith BAH, Linde MH, Coles GL, Dalton GN (2022). Anti-GD2 synergizes with CD47 blockade to mediate tumor eradication. Nature Medicine.

[25] Kolter T, Proia RL, Sandhoff K (2002). Combinatorial ganglioside biosynthesis. J Biol Chem.

[26] Furukawa K, Takamiya K, Furukawa K (2002). Beta1,4-N-acetylgalactosaminyltransferase-GM2/GD2 synthase: a key enzyme to control the synthesis of brain-enriched complex gangliosides. Biochim Biophys Acta.

[27] Hung J-T, Yu AL (2019). Chapter 4 - GD2-Targeted Immunotherapy of Neuroblastoma. In: Ray SK, editor. Neuroblastoma: Academic Press.

[28] Nazha B, Inal C, Owonikoko TK (2020). Disialoganglioside GD2 Expression in Solid Tumors and Role as a Target for Cancer Therapy. Front Oncol.

[29] Svennerholm L, Boström K, Fredman P, Jungbjer B, Lekman A, Månsson JE (1994). Gangliosides and allied glycosphingolipids in human peripheral nerve and spinal cord. Biochim Biophys Acta.

[30] Uttenreuther-Fischer MM, Huang CS, Reisfeld RA, Yu AL (1995). Pharmacokinetics of anti-ganglioside GD2 mAb 14G2a in a phase I trial in pediatric cancer patients. Cancer Immunol Immunother.

[31] Murray JL, Cunningham JE, Brewer H, Mujoo K, Zukiwski AA, Podoloff DA (1994). Phase I trial of murine monoclonal antibody 14G2a administered by prolonged intravenous infusion in patients with neuroectodermal tumors. J Clin Oncol.

[32] Yu AL, Uttenreuther-Fischer MM, Huang CS, Tsui CC, Gillies SD, Reisfeld RA (1998). Phase I trial of a human-mouse chimeric anti-disialoganglioside monoclonal antibody ch14.18 in patients with refractory neuroblastoma and osteosarcoma. J Clin Oncol.

[33] Navid F, Sondel PM, Barfield R, Shulkin BL, Kaufman RA, Allay JA (2014). Phase I trial of a novel anti-GD2 monoclonal antibody, Hu14.18K322A, designed to decrease toxicity in children with refractory or recurrent neuroblastoma. J Clin Oncol.

[34] Yoshida H, Koodie L, Jacobsen K, Hanzawa K, Miyamoto Y, Yamamoto M (2020). B4GALNT1 induces angiogenesis, anchorage independence growth and motility, and promotes tumorigenesis in melanoma by induction of ganglioside GM2/GD2. Sci Rep.

[35] Luu HH, Kang Q, Park JK, Si W, Luo Q, Jiang W (2005). An Orthotopic Model of Human Osteosarcoma Growth and Spontaneous Pulmonary Metastasis. Clinical & Experimental Metastasis.

[36] Crocker PR, Paulson JC, Varki A (2007). Siglecs and their roles in the immune system. Nat Rev Immunol.

[37] Avril T, Floyd H, Lopez F, Vivier E, Crocker PR (2004). The membrane-proximal immunoreceptor tyrosine-based inhibitory motif is critical for the inhibitory signaling mediated by Siglecs-7 and -9, CD33-related Siglecs expressed on human monocytes and NK cells. J Immunol.

[38] Li T, Xu B, Li W, Cheng X, Tantai W, Zheng H (2024). Allosteric inhibitor of SHP2 enhances macrophage endocytosis and bacteria elimination by increasing caveolae activation and protects against bacterial sepsis. Pharmacol Res.

[39] Hingorani P, Krailo M, Buxton A, Hutson P, Sondel PM, Diccianni M (2022). Phase 2 study of anti-disialoganglioside antibody, dinutuximab, in combination with GM-CSF in patients with recurrent osteosarcoma: A report from the Children's Oncology Group. Eur J Cancer.

[40] Li L, Rong G, Gao X, Cheng Y, Sun Z, Cai X (2025). Bone-Targeted Fluoropeptide Nanoparticle Inhibits NF-κB Signaling to Treat Osteosarcoma and Tumor-Induced Bone Destruction. Advanced Science.

[41] He Y, Cheng D, Lian C, Liu Y, Luo W, Wang Y (2021). Serglycin induces osteoclastogenesis and promotes tumor growth in giant cell tumor of bone. Cell Death Dis.

[42] He Y, Luo W, Liu Y, Wang Y, Ma C, Wu Q (2022). IL-20RB mediates tumoral response to osteoclastic niches and promotes bone metastasis of lung cancer. J Clin Invest.

[43] Christofides A, Katopodi XL, Cao C, Karagkouni D, Aliazis K, Yenyuwadee S (2023). SHP-2 and PD-1-SHP-2 signaling regulate myeloid cell differentiation and antitumor responses. Nat Immunol.

[44] Guide for the Care and Use of the Nude (Thymus-Deficient) Mouse in Biomedical Research Washington, DC: The National Academies Press. 1976.

[45] Yu J, Fu L, Wu R, Che L, Liu G, Ran Q (2025). Immunocytes in the tumor microenvironment: recent updates and interconnections. Front Immunol.

[46] Eisel D, Das K, Dickes E, König R, Osen W, Eichmüller SB (2019). Cognate Interaction With CD4(+) T Cells Instructs Tumor-Associated Macrophages to Acquire M1-Like Phenotype. Front Immunol.

