# Supplementary material for: GD2-mediated impairment of macrophage phagocytosis drives pulmonary metastasis in osteosarcoma
Source: Theranostics. 2025 Jun 20;15(15):7454–66. doi: 10.7150/thno.113887 (PMC12315822; doi:10.7150/thno.113887)
Supplement: Supplementary file 1 — Supplementary figures. [file thnov15p7454s1.pdf]

GD2-mediated impairment of macrophage phagocytosis drives pulmonary metastasis in osteosarcoma

Yunfei He[1,2,#], Peng Yang[1,#], Peng Ding[3,#], Zeng Zhang[3,#], Jichuan Wu[4], Chunjie Wang[5], Guanghui Hou[5], Jun Ge[1], Quan Zhou[1], Zhuorun Song[1], Huilin Yang[1,*], Tao Liu[1,*], Shunyi Lu[1,*]

Affiliations

[1] Department of Orthopedics, The First Affiliated Hospital of Soochow University, Suzhou 215006, China

[2] Institutes for Translational Medicine, The First Affiliated Hospital of Soochow University, Suzhou, 215123, China

[3] Department of Orthopedics, Shanghai Sixth People's Hospital Affiliated to Shanghai Jiao Tong University School of Medicine, Shanghai, 200233, China.

[4] Biomedical Polymers laboratory, college of chemistry chemical engineering and Materials Science and State Key laboratory of Radiation Medicine and Protection, Soochow University, Suzhou 215123, China

[5] Institute of Functional Nano & Soft Materials (FUNSOM), Jiangsu Key Laboratory for Carbon-Based Functional Materials & Devices, Soochow University, Suzhou, 215123, China

* Corresponding author. Email: hlyang@suda.edu.cn; liutao8250@suda.edu.cn; lushunyi@suda.edu.cn.

# These authors contributed equally to this work.

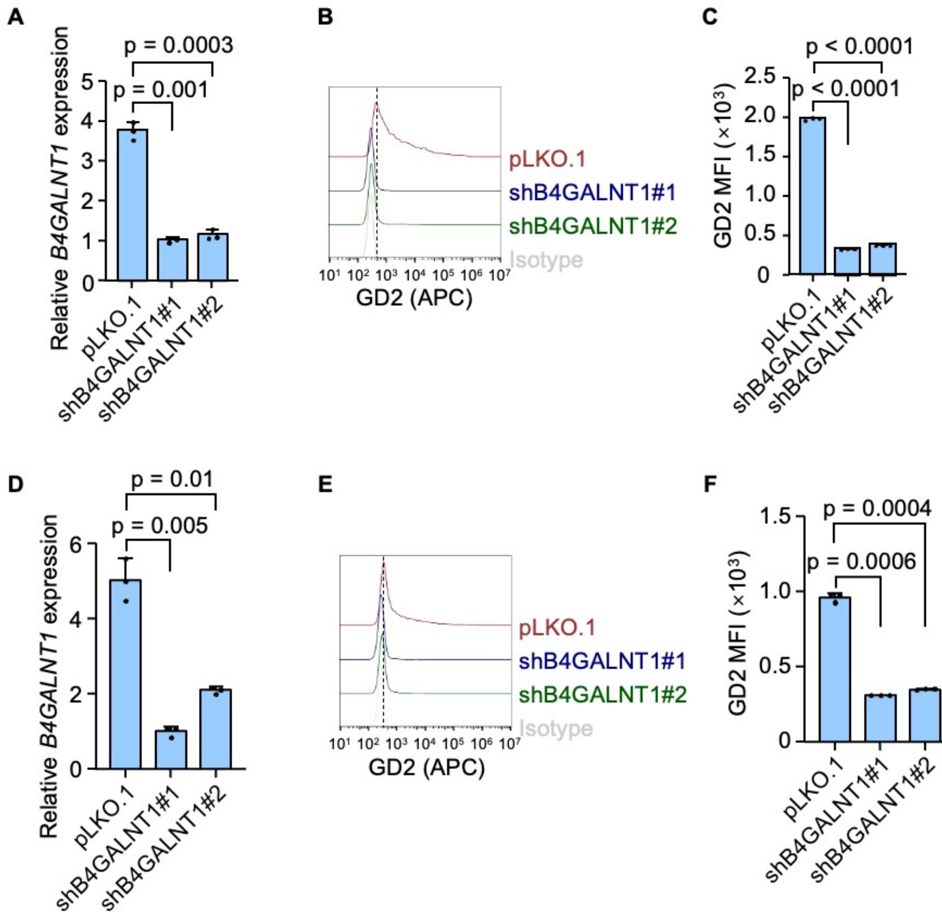

Figure S1. GD2 depletion in different osteosarcoma cell lines. (**A-C**) GD2 depletion in U2OS. Shown are quantitative PCR analysis to validate *B4GALNT1* knockdown (**A**), Flow cytometric analysis of GD2 expression in U2OS with or without *B4GALNT1* knockdown (**B**) and quantitation of GD2 mean fluorescence intensity (MFI) (**C**). (**D-E**) GD2 depletion in 143B. Shown are quantitative PCR analysis to validate *B4GALNT1* knockdown (**D**), Flow cytometric analysis of GD2 expression in U2OS with or without *B4GALNT1* knockdown (**E**) and quantitation of GD2 mean fluorescence intensity (MFI) (**F**). P values were obtained by 2-tailed unpaired t test (**A**, **C**, **D** and **F**). Data are represented as mean ± SD.

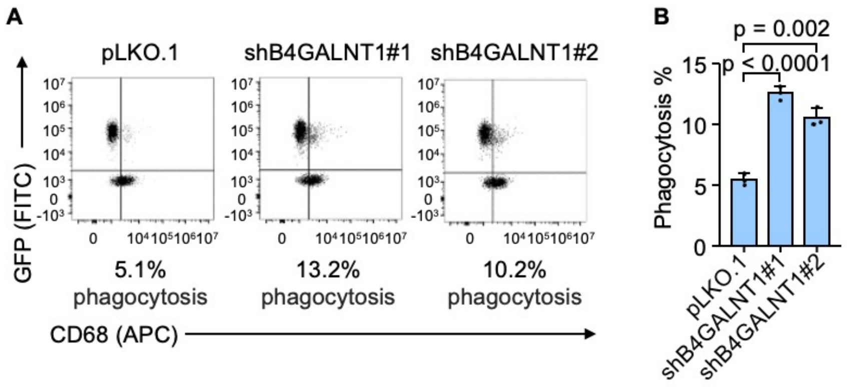

Figure S2. GD2 suppresses phagocytosis of osteosarcoma cells. (**A**, **B**) Representative flow cytometry plots depicting the phagocytosis of GFP-labeled U2OS cells (with or without *B4GALNT1* knockdown) co-cultured with THP-1 derived macrophages (**A**) and flow-cytometry-based quantification of phagocytosis of U2OS in the presence of THP-1 derived macrophages (**B**). P values were obtained by 2-tailed unpaired t test (**B**). Data are represented as mean ± SD.

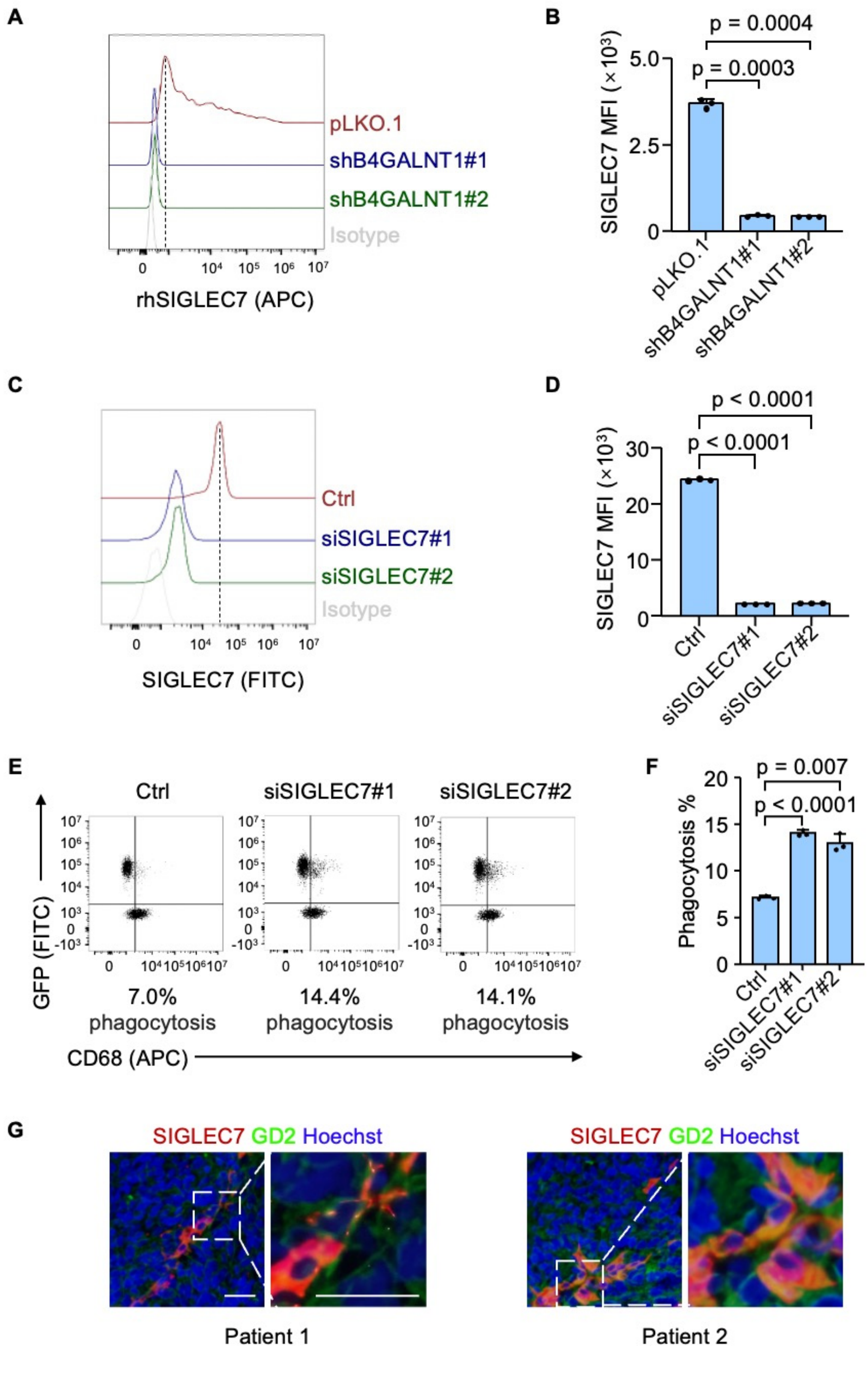

Figure S3. GD2 functions by interacting with SIGLECs. (**A**) Flow cytometric histograms of U2OS (with or without *B4GALNT1* knockdown) stained with recombinant human SIGLEC7. (**B**) Quantitation of SIGLEC7 mean fluorescence intensity (MFI) in panel (**A**). (**C**) Flow cytometric analysis of SIGLEC7 expression in THP-1 (with or without *SIGLEC7*

knockdown). (**D**) Quantitation of SIGLEC7 mean fluorescence intensity (MFI) in panel (**C**). (**E**, **F**) Representative flow cytometry plots depicting the phagocytosis of GFP-labeled U2OS cells co-cultured with THP-1 derived macrophages (with or without *SIGLEC7* knockdown) (**E**) and flow-cytometry-based quantification of phagocytosis of U2OS in the presence of mouse bone marrow derived macrophages(**F**). (**G**) SIGLEC7 and GD2 immunostaining of lung metastases from osteosarcoma patients. Scale bars: 25 μm. P values were obtained by 2-tailed unpaired t test (**B**, **D** and **F**). Data are represented as mean ± SD.

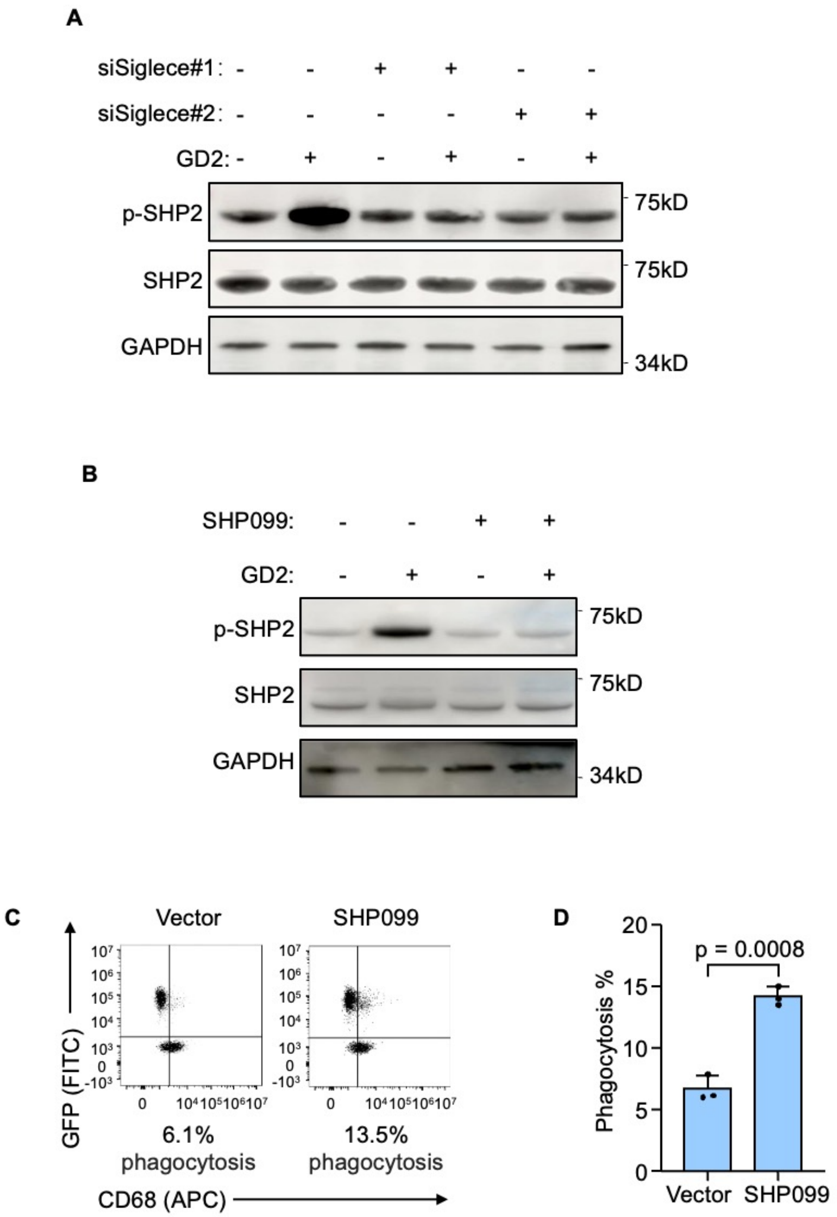

Figure S4. GD2 activates SH2-containing protein tyrosine phosphatase 2. (**A**) Western blot analysis of phosphorylated SHP2 protein level in mouse bone marrow derived macrophages (with or without *Siglece* knockdown) after treated with GD2. (**B**) Western blot analysis of phosphorylated SHP2 protein level in THP-1 derived macrophages after treated with GD2 and/or SHP099. (**C**, **D**) Representative flow cytometry plots depicting the phagocytosis of GFP-labeled U2OS cells co-cultured with THP-1 derived macrophages (with or without SHP099 treatment) (**B**) and flow-cytometry-based quantification of phagocytosis of U2OS in the presence of mouse bone marrow derived macrophages (**C**).

P values were obtained by 2-tailed unpaired t test (**D**). Data are represented as mean ± SD.

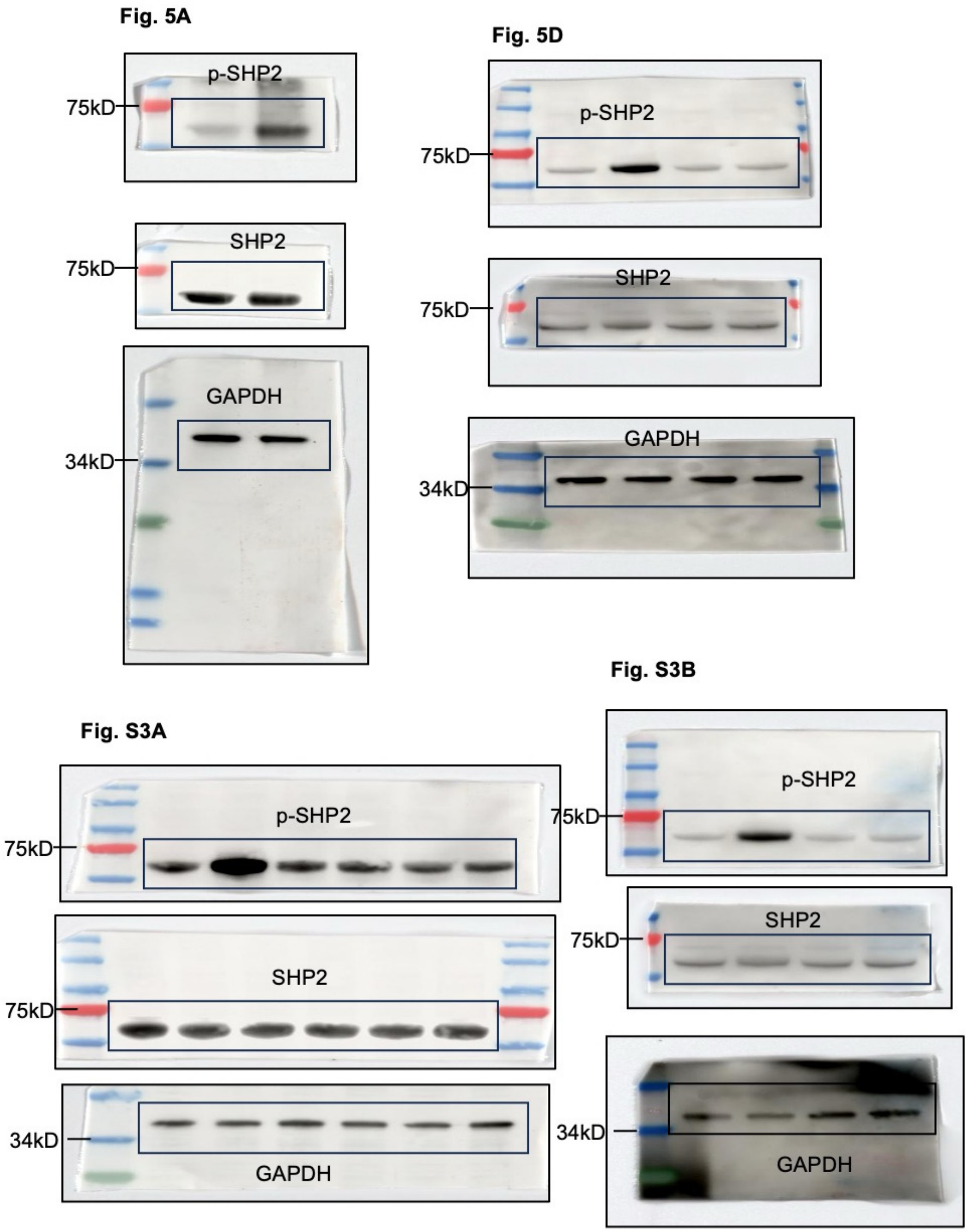

Figure S5. Uncropped gels.
